# Data mining identifies novel RNA-binding proteins involved in colon and rectal carcinomas

**DOI:** 10.3389/fcell.2023.1088057

**Published:** 2023-06-13

**Authors:** Jennyfer M. García-Cárdenas, Isaac Armendáriz-Castillo, Nathali García-Cárdenas, David Pesantez-Coronel, Andrés López-Cortés, Alberto Indacochea, Santiago Guerrero

**Affiliations:** ^1^ Laboratorio de Ciencia de Datos Biomédicos, Escuela de Medicina, Facultad de Ciencias Médicas de la Salud y de la Vida, Universidad Internacional del Ecuador, Quito, Ecuador; ^2^ Latin American Network for the Implementation and Validation of Clinical Pharmacogenomics Guidelines (RELIVAF-CYTED), Madrid, Spain; ^3^ Facultad de Ingenierías y Ciencias Aplicadas, Universidad Internacional SEK, Quito, Ecuador; ^4^ Instituto Nacional de Investigación en Salud Pública, Quito, Ecuador; ^5^ Medical Oncology Department Hospital Clinic and Translational Genomics and Targeted Therapies in Solid Tumors, IDIBAPS, Barcelona, Spain; ^6^ Cancer Research Group (CRG), Faculty of Medicine, Universidad de Las Américas, Quito, Ecuador

**Keywords:** RNA-binding proteins (RBPs), colorectal adenocarcinoma (COREAD), rectum, colon, biomarkers, cancer, data mining, multi-omics

## Abstract

Colorectal adenocarcinoma (COREAD) is the second most deadly cancer and third most frequently encountered malignancy worldwide. Despite efforts in molecular subtyping and subsequent personalized COREAD treatments, multidisciplinary evidence suggests separating COREAD into colon cancer (COAD) and rectal cancer (READ). This new perspective could improve diagnosis and treatment of both carcinomas. RNA-binding proteins (RBPs), as critical regulators of every hallmark of cancer, could fulfill the need to identify sensitive biomarkers for COAD and READ separately. To detect new RBPs involved in COAD and READ progression, here we used a multidata integration strategy to prioritize tumorigenic RBPs. We analyzed and integrated 1) RBPs genomic and transcriptomic alterations from 488 COAD and 155 READ patients, 2) ∼ 10,000 raw associations between RBPs and cancer genes, 3) ∼ 15,000 immunostainings, and 4) loss-of-function screens performed in 102 COREAD cell lines. Thus, we unraveled new putative roles of NOP56, RBM12, NAT10, FKBP1A, EMG1, and CSE1L in COAD and READ progression. Interestingly, FKBP1A and EMG1 have never been related with any of these carcinomas but presented tumorigenic features in other cancer types. Subsequent survival analyses highlighted the clinical relevance of FKBP1A, NOP56, and NAT10 mRNA expression to predict poor prognosis in COREAD and COAD patients. Further research should be performed to validate their clinical potential and to elucidate their molecular mechanisms underlying these malignancies.

## Introduction

Colorectal adenocarcinoma (COREAD) has been ranked as the second most deadly cancer and the third most common malignancy worldwide with an estimated 1.9 million cases and 0.9 million deaths in 2020 (Xi and Xu, 2021). Over the past 10 years, significant advances were achieved in personalized treatments for COREAD patients based on the molecular subtyping (Cohen et al., 2020; López-Cortés et al., 2020; Assis et al., 2022). For example, metastatic COREAD patients, harboring *BRAF* ^V600E^ mutation, have now better treatment options (Mauri et al., 2021). Despite these efforts, molecular subtyping has been insufficient to address the heterogeneity of colon and rectal tumors (Cohen et al., 2020; Liu Z. et al., 2021; Assis et al., 2022). In that context, Paschke *et al.*, after analyzing ∼2000 publications and the results of two large clinical trials, suggested stopping using the term COREAD and started separating into two different tumor identities: colon cancer (COAD) and rectal cancer (READ). Paschke *et al.*, reached this conclusion by describing obvious differences between COAD and READ concerning molecular carcinogenesis, pathology, surgical topography and procedures, and multimodal treatment. This new perspective could improve the identification of new biomarkers and therapeutic targets for both types of cancer (Paschke et al., 2018).

A new emerging understanding of RNA-binding proteins (RBPs) have addressed them as critical modulators of every hallmark of cancer (Abdel-Wahab and Gebauer, 2018; García-Cárdenas et al., 2019; Hanahan, 2022). RBPs can modulate the expression levels of oncogenes and tumor suppressors (Hentze et al., 2018; García-Cárdenas et al., 2019; Kang et al., 2020) by controlling all aspects of their mRNA processing and metabolism, such as capping, polyadenylation, alternative splicing, subcellular localization, nucleocytoplasmic transport, stability, and degradation (Hentze et al., 2018; García-Cárdenas et al., 2019; Kang et al., 2020; Mestre-Farràs et al., 2022). Thus, identification of tumorigenic RBPs could fulfill the need to discover more accurate and sensitive therapeutic targets for COAD and READ (Cohen et al., 2020; Liu Z. et al., 2021; Assis et al., 2022).

In that respect, we previously performed a literature review to identify RBPs implicated in COREAD (García-Cárdenas et al., 2019). As a result, we found 35 RBPs (out of 1,392 described by Hentze et al., 2018) involved in different aspects of COREAD progression, such as angiogenesis, metastasis, or chemotherapy resistance (Hentze et al., 2018). We also showed that these RBPs are implicated in a complex interconnected network where a single RBP can bind to thousands of RNAs. For instance, ELAVL1 targets 21,578 RNAs, whereas KHDRBS1 interacts with 962. These results pointed out the potential of RBPs to regulate cancerous cellular processes and thereby to be used as COAD or READ biomarkers.

In extending the scope of our previous work and discovering new RBPs involved in COAD and READ separately, here we used our previously published multidata integration strategy to prioritized tumorigenic RBPs (García-Cárdenas et al., 2022). Thus, we assembled data from several resources: The Cancer Genome Atlas (Tomczak et al., 2015), The Human Protein Atlas (Pontén et al., 2008), STRING (Szklarczyk et al., 2019), Depmap (Yu et al., 2016) and HumanNet (Kim et al., 2022), and revealed new RBPs associated with both types of cancer. Our results provide a better understanding of COAD and READ biology and potentially unveil new targets for cancer therapy and prognostic biomarkers.

## Methods

### Gene sets

Hentze et al., compiled all published RNA interactomes and stringently curated a list of 1,393 RBPs (Hentze et al., 2018). After checking for new annotations using Ensembl (http://www.ensembl.org), we found one duplicate (ENSG00000100101 and ENSG00000273899, both corresponded to NOL12), leaving a final list of 1,392 RBPs. The cancer driver genes (*n* = 2,372) were retrieved from the Network of Cancer Genes 7.0 (NCG7, http://ncg.kcl.ac.uk/) (Dressler et al., 2022) and filtered by COREAD genes (*n* = 156) (Supplementary Table S1).

### Genomic and transcriptomic data exploration

The cBioPortal for Cancer Genomics (https://www.cbioportal.org; accessed on 04 March 2022) was used to analyze and retrieve genomic and transcriptomic alterations of RBPs from datasets that clinically differentiate COAD and READ. Specifically, we used the Colorectal Adenocarcinoma dataset (TCGA, PanCancer Atlas; Hoadley et al., 2018) which has 378 COAD patients and 155 READ patients. We also analyzed the Colon Cancer study (CPTAC-2 Prospective; Vasaikar et al., 2019) which has 110 complete COAD samples. To compare the aforementioned gene sets, genomic and transcriptomic alterations were corrected by the number of patients. As COAD and READ sets have different number of patients, we divided the number of alterations per RBP by number of patients, i.e., the mean of genomic and transcriptomic alterations per RBP. A Mann–Whitney *U* test was applied when comparing clinical characteristics or genomic and transcriptomic alterations between gene sets (colon vs. rectum) and within each group (COAD and READ stages and subtypes) (Supplementary Tables S2–S7). Additionally, mRNA Z-scores of aberrantly expressed RBPs in COAD and READ were collected and compared using a Mann–Whitney *U* test (Supplementary Tables S8, S9). A z-score of < −2 or >2 (*p*-value = <0.05; confidence level 95%) was used as the criteria for RBPs being determined as down/upregulated, respectively.

### Gene network construction

Experimental and database interactions between RBPs (*n* = 1,392) and COREAD proteins (*n* = 156) (Supplementary Table S10), having an interaction score of 0.9 (highest confidence), were predicted with the STRING database (Szklarczyk et al., 2015; Repana et al., 2019). Then, the network was visualized using the Cytoscape 3.9.1 (Seattle, USA) platform (Shannon et al., 2003).

### Protein expression analysis

Protein immunohistochemical levels were extracted from The Human Protein Atlas version 21.1 (https://www.proteinatlas.org; accessed on 15 March 2022) (Uhlén et al., 2015; Thul et al., 2017; Uhlen et al., 2017). We obtained protein expression levels (not detected, low, medium, and high) for 608 RBPs in COAD and 609 RBPs in READ tissues. Protein expression levels of normal tissues were taken from glandular cells, while a consensus level was manually generated for COAD and READ tissues (Supplementary Tables S11, S12) based on the expression levels. Both normal and tumor tissues had antibody validation parameters. Only enhanced and supported parameters were considered for this analysis.

### Cancer genetic dependency analysis

RBPs cancer dependency scores from CERES (Meyers et al., 2017) (1,341 available RBPs) and DEMETER2 (Tsherniak et al., 2017; McFarland et al., 2018) (1,255 available RBPs) were obtained from the Dependency Map (DepMap) portal (https://depmap.org/portal) (Yu et al., 2016). A score of 0 = not essential gene for cell survival, whereas a score of −1 corresponds to the median of all common essential genes, i.e., genes whose principal cellular processes are involved in fundamental cell survival pathways. These scores were calculated from gene knock-out (CERES) and knock-down (Demeter) experiments performed in cancer cell lines. CERES reported data from 42 COAD cell lines and three READ cell lines, while DEMETER2 obtained data from 47 COAD cell lines and 10 READ cell lines (Supplementary Tables S13–S16).

### Integrative gene network

The prioritized RBPs for COAD and READ were integrated into a disease gene network by using the HumanNet XC (functional gene network extended network by co-citation) latest version (v3 software) (https://www.inetbio.org/humannet) (Kim et al., 2022) and visualized through Cytoscape 3.9.1 (Shannon et al., 2003) (Supplementary Tables S17, S18).

### Clinical analysis

The TCGA, PanCancer Atlas (Hoadley et al., 2018) colorectal cancer database was inspected for mRNA expression of prioritized RBPs in colon and rectum patients. Probability of overall survival (OS) and disease-free survival (DFS) were calculated. Curves were obtained by dividing samples in two groups using median z-score as a cutoff in COREAD, COAD, and READ patients. These two groups represent 1) patients with RBP mRNA upregulation (blue lines) and 2) patients with RBP mRNA downregulation (red lines). Differences between groups were calculated using log rank test. Graphical representations and statistical analysis were performed with IBM SPSS, version 22. Only significant comparisons with an N > 20 per group is shown.

## Results

### Multidata integration strategy to prioritized tumorigenic RNA-binding proteins

**FIGURE 1:**
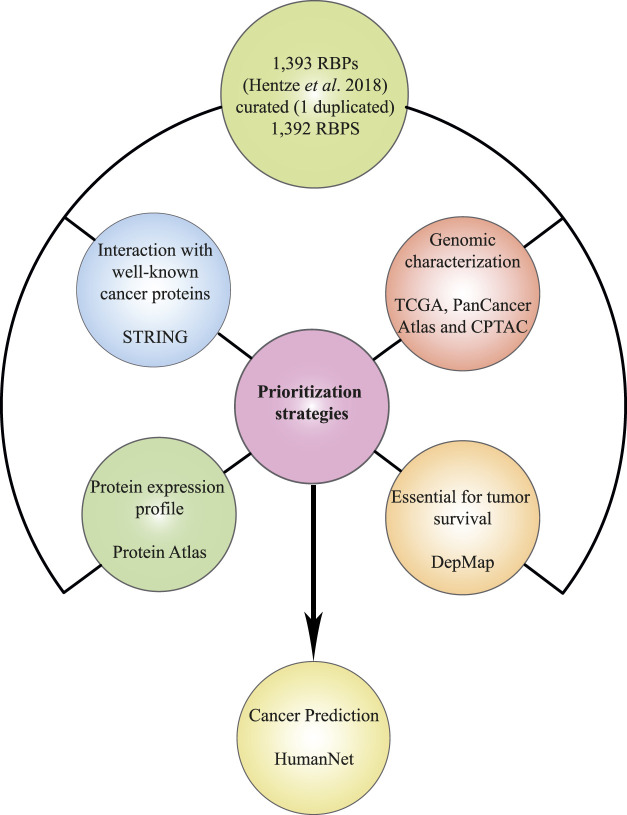
Schematic representation of the data mining strategy. All databases interrogated for prioritization: The Cancer Genome Atlas (Tomczak et al., 2015), The Human Protein Atlas (Pontén et al., 2008), STRING (Szklarczyk et al., 2019), and DepMap (Yu et al., 2016), and further cancer association analysis (HumanNet; Kim et al., 2022) are depicted in the multidata integration workflow.

We previously published a multidata integration strategy that allowed us to identify PUF60 and SF3A3 as new spliceosome-related breast cancer RBPs (García-Cárdenas et al., 2022). In this work, we used the same strategy to prioritize tumorigenic RBPs that could be used as COAD or READ biomarkers. First, we performed individual analysis of several databases such as The Cancer Genome Atlas (Tomczak et al., 2015), The Human Protein Atlas (Pontén et al., 2008), STRING (Szklarczyk et al., 2019), and Depmap (Yu et al., 2016) to identify RBPs with different cancer-related characteristics: 1) high genomic and transcriptomic alterations, 2) interactions with well-known cancer proteins, 3) aberrant protein expression levels compared with normal tissues, and 4) essential for tumor survival. Next, we performed a rigorous analysis to detect RBPs with all the aforementioned attributes, and thereby prioritizing potential COAD and READ RBPs. Finally, we predicted how these prioritized RBPs are correlated with cancer phenotypes using the HumanNet (Kim et al., 2022) database (Figure 1).

### Detection of highly altered RNA-binding proteins in colon and rectal tumors

**FIGURE 2:**
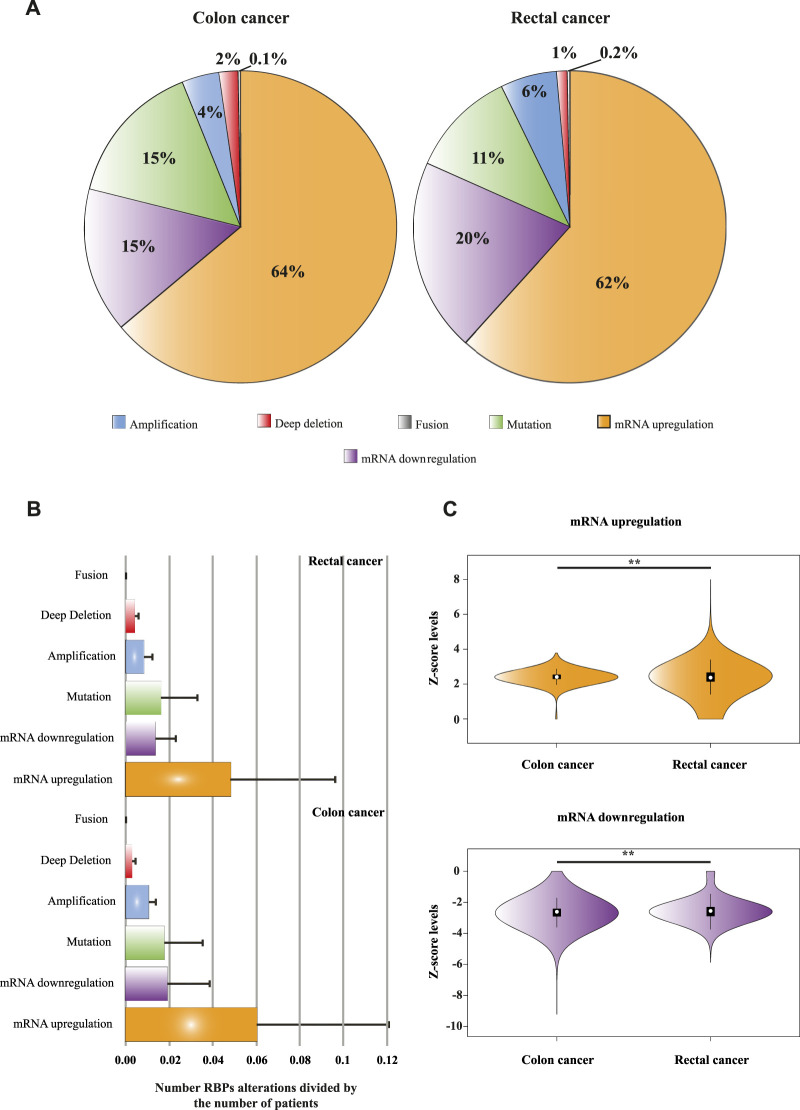
Genome and transcriptome alterations of RNA Binding Proteins. **(A)** A pie chart illustrating different types of RBPs genomic and transcriptomic alterations and their percentages in colon and rectal tumors. Data was obtained from Colorectal Adenocarcinoma study (TCGA, PanCancer Atlas; Hoadley et al., 2018) and Colon Cancer study (CPTAC-2 Prospective; Vasaikar et al., 2019). **(B)** Number of RBPs alterations (corrected by number of patients, arbitrary units) separated by type. A Mann–Whitney *U* test was performed to compare alterations between datasets (COAD vs. READ alterations). All possible comparisons between sets presented significant statistically differences (*p* < 0.001) except mutations and fusions; ns = not significant. **(C)** Violin plots portraying the differences of mRNA levels (Z-scores) between colon and rectal tumors, a Mann–Whitney *U* test was performed to compare these data sets. ** = high statistically significant difference.

To globally determine the genomic and transcriptomic alterations of RBPs in COAD and READ patients, we interrogated two independent datasets, TCGA, PanCancer Atlas (Hoadley et al., 2018) and Colon Cancer study CPTAC-2 Prospective (Vasaikar et al., 2019) including *in toto* 488 COAD and 155 READ patients. First, we compared RBPs (*n* = 1,392) genomic and transcriptomic alterations between COAD and READ. Once we corrected by the number of patients (i.e., mean of genomic and transcriptomic alterations per RBP), we found significant differences (*p* < 0.001) between COAD and READ. In Figure 2A is depicted the percentages of each alteration, where mRNA upregulation accounted for most of the genomic and transcriptomic modifications in COAD and READ. In colon tumors we found that mRNA downregulation and mutations presented equal percentages (15%), whereas mRNA downregulation (20%) occupied the second place followed by mutations (11%) in READ.

Although the pattern of RBPs genomic and transcriptomic alterations in COAD and READ are similar (Figure 2A), when they are analyzed separately, significant differences were found (*p* < 0.001) except for mutations and fusions in COAD vs. READ (Figure 2B; Supplementary Tables S2, S3). Even though RBPs mRNA upregulations account for 64% in COAD vs. 62% in READ, mRNA Z-scores were higher in READ than in COAD (*p* < 0.001) (Figure 2C; Supplementary Tables S8, S9). The contrary is appreciated when analyzing mRNA downregulation, COAD RBPs z-scores distribution shows a wider mRNA downregulation compared to READ RBPs (*p* < 0.001) (Figure 2C).

To identify RBPs involved in tumor progression or suppression, RBPs genomic and transcriptomic alterations categories were classified accordingly. In malignant cells, mRNA upregulation and genomic amplifications are related to tumor progressors, while mRNA downregulation and genomic deletions are connected with suppressors (Wurth et al., 2016; Mestre-Farràs et al., 2022). Gene fusion and mutations have been detected in both tumor progressors and suppressors. Based on these principles, we listed the most frequently altered RBPs in COAD and READ (Table 1, Supplementary Tables S2, S3). As expected, most of them have already been related to COAD or READ, and thereby validating our strategy (Table 1). Interestingly, STAU1 was the most altered RBP in both carcinomas, and yet it has never been correlated with COAD or READ; however, it has been associated with prostate cancer (Marcellus et al., 2021). Similarly, some progressors (CHMP4B, CSTF1, and LSM14B) and suppressors RBPs (ATP5F1A, GTF2E2, RTF1, ELAC2, LRRC47, and MRM3) have never been associated with COAD or READ, but present oncogenic properties in other cancer types (Table 1, Supplementary Tables S2, S3). Worthy of note, we also identified RBPs that are unique for each cancer type and others (e.g., CSTF1) that have been never related to cancer (Table 1).

**TABLE 1:** Most frequently altered RNA-binding proteins in colon and rectal cancer. Data was obtained from Colorectal Adenocarcinoma study (TCGA, PanCancer Atlas; Hoadley et al., 2018) and Colon Cancer study (CPTAC-2 Prospective; Vasaikar et al., 2019).

Genomic and transcriptomic alterations	Protein name	COAD (C) or READ (R)/Number of alterations	Known COAD or READ molecular and cellular functions	Related to other cancer types/
upregulation + fusion + mutations	STAU1	C/295	No	Yes Marcellus et al. (2021)
		R/291		
	YTHDF1	C/291	Yes. YTHDF1 Regulates Tumorigenicity in Human Colorectal Carcinoma Bai et al. (2019); Chen P et al. (2021); Yan et al. (2021)	Yes Cao et al. (2017)
		R/146		
	CHMP4B	C/272	No	Yes Hu et al. (2015)
		R/127		
	DDX27	C/266	Yes. DDX27 promotes Colorectal cancer growth and metastasis Tang et al. (2018)	Yes Li et al. (2021)
		R/138		
	CSTF1	R/128	No	No
	DIDO1	C/250	Yes. DIDO1 promotes carcinoma progression Sillars-Hardebol et al. (2012)	Yes Forghanifard et al. (2020)
		R/117		
	RBM39	C/248	Yes. RBM39 promotes carcinoma progression Sillars-Hardebol et al. (2012)	Yes Xu et al. (2022)
	EIF2S2	C/247	Yes. EIF2S2 may promote glycolysis in CRC Yang et al. (2021)	Yes Ji et al. (2021)
		R/121		
	RPRD1B	C/245	Yes. Overexpression of RPRD1B confers colorectal cancer sensitivity to fluorouracil (Kuang et al., 2018)	Yes Wen et al. (2020)
	LSM14B	C/244	No	Yes Ta et al. (2021)
		R/125		
	NCOA5	C/243	Yes. NCOA5 promotes proliferation, migration and invasion of colorectal cancer cells Sun et al. (2017)	Yes Tan et al. (2021)
		R/127		
	PRPF6	R/114	Yes. PRPF6 is essential for tumor growth Adler et al. (2014)	Yes Liu W et al. (2021)
Suppressors Deep deletion + mRNA downregulation + fusion + mutations	CCAR2	C/140	Yes. CCAR2 mediates colon cancer progression Kim et al. (2018)	Yes Chen L et al. (2021)
		R/76		
	NARS	C/116	Yes. Novel genes associated with colorectal cancer Eldai et al. (2013)	No
	FXR2	C/100	Yes. Differentially Expressed Profiles of mRNA N6-Methyladenosine in Colorectal Cancer Li N et al. (2022)	No
		R/46		
	ATP5F1A	C/99	No	Yes Feichtinger et al. (2018)
		R/60		
	SYNE1	C/94	Yes. SYNE1 mutations are related with worse survival outcomes Zhou et al. (2020)	Yes Qu et al. (2021)
		R/31		
	GTF2E2	C/84	No	Yes Bi et al. (2021)
		R/42		
	ALKBH5	C/76	Yes. Related to tumor immunity in colon adenocarcinoma Yan et al. (2021)	Yes Guo et al. (2020)
	NCBP3	C/71	No	No
	RTF1	C/71	No	No
	ELAC2	C/63	No	Yes Noda et al. (2006)
		R/60		
	LRRC47	R/37	No	Yes Mu et al. (2022)
	YWHAE	R/31	Yes. YWHAE mediated the function of miR-6778–5p in the proliferation of colorectal cancer cells Li et al. (2019)	Yes Yang et al. (2019)
	MRM3	R/30	No	Yes Schmidlin et al. (2016)
	DHX33	R/29	Yes. It promotes colon cancer development downstream of Wnt signaling Zhu et al. (2020)	Yes Tian et al. (2016)

**FIGURE 3:**
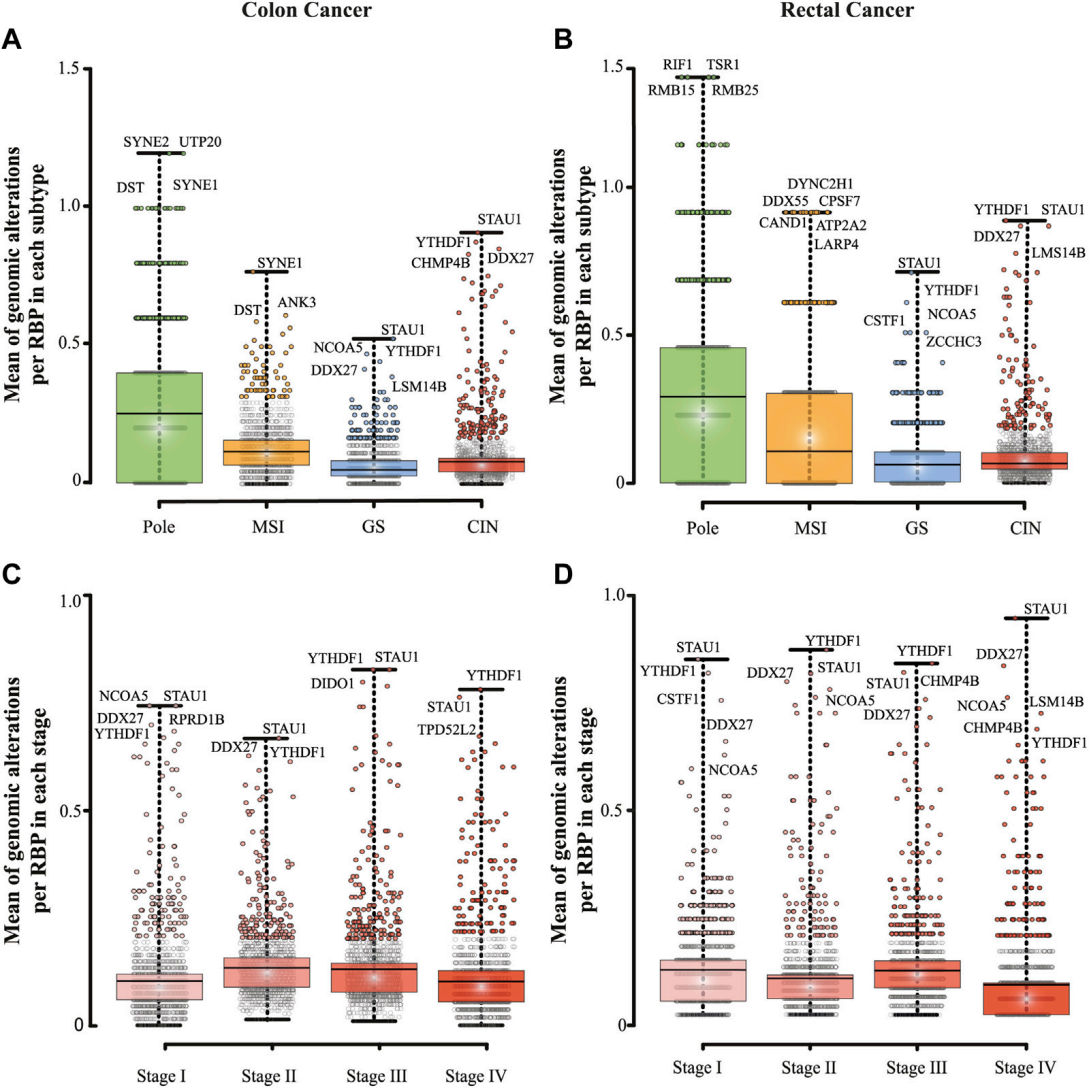
Detection of highly altered RNA-binding proteins in colon and rectal carcinomas. RBPs genomic and transcriptomic alterations per subtype **(A, B)** and stage **(C, D)** are depicted in boxplots. Data was obtained from Colorectal Adenocarcinoma study (TCGA, PanCancer Atlas; Hoadley et al., 2018) and Colon Cancer study (CPTAC-2 Prospective; Vasaikar et al., 2019). The number of RBPs alterations was divided by the number of patients in each set of data. A Mann–Whitney *U* test was performed to compare genomic and transcriptomic alterations between sets. All possible comparisons between colon and rectum and within each data set in both carcinomas presented high statistically significant difference (*p* < 0.001), except for GS subtype when comparing colon vs. rectum, stage 1 vs. stage 4 (in colon cancer), and stage 1 vs. 3 (in rectal cancer); ns = not significant.

We, next, determined genomic and transcriptomic alterations of RBPs by subtypes, pole (polymerase ε), microsatellite instability (MSI), genomically stable (GS), and chromosomal instability (CIN) (Figures 3A,B; Table 2; Supplementary Tables S4, S5) and stages (Stage I to IV, Table 2; Figures 3C,D; Supplementary Tables S6, S7). We found statistically significant differences between all subtypes in both carcinomas (Mann–Whitney U, *p* < 0.001; Figures 3A,B). Pole was the most altered subtype, followed by MSI, CIN, and GS in both malignancies (Figures 3A,B). When comparing subtypes between COAD and READ, we also found statistically significant differences except for COAD GS vs. READ GS subtype (Mann–Whitney U, *p* < 0.001; Figures 3A,B). In COAD, we also observed equally altered RBPs among subtypes. For example, DST and SYNE1 were highly altered in Pole and MSI, while DDX27, STAU1, and YTHDF1 in GS and CIN. It is important to mention that some of these RBPs were subtype specific such as SYNE2 and UTP20 for Pole, and ANK3 for MSI (Figure 3A). Similarly, we found subtype specific RBPs within READ subtypes that have never related to COREAD. For instance, DYNC2H1, DDX55, CPSF7, CAND1 and LARP4 were found in MSI subtype, TSR1 in Pole, ZCCHC3 in GS, and LSM14B in CIN (Figure 3B).

**TABLE 2:** Mean of genomic and transcriptomic alterations per stage and subtypes and sample number in COAD and READ patients.

Subtypes	Colon (number of patients/Mean)	Rectum (number of patients/Mean)
Pole	55/0.252	4/0.319
MSI	44/0.128	3/0.122
CIN	36/0.092	9/0.097
GS	202/0.061	99/0.076
**Stages**		
I	78/0.104	29/0.113
II	186/0.135	50/0.091
III	150/0.131	44/0.112
IV	62/0.103	25/0.074

We also detected statistically significant differences between all stages in both cancer types (Mann–Whitney U, *p* < 0.001; Figures 3C,D). In COAD the highest mean of RBPs genomic and transcriptomic alterations were detected in stage II, while in READ they were found in stage I (Figures 3C,D; Table 2). Interestingly, stage IV showed the lowest number of RBPs alterations in both cancers. Despite these differences, STAU1 and YTHDF1 were constantly altered in all stages. This was also observed in GS and CIN subtypes (Figures 3A,B; Table 2). Contrary to STAU1, YTHDF1 has previously been associated with COREAD development (Bai et al., 2019; Chen P. et al., 2021; Yan et al., 2021), showing the potential of STAU1 to be involved in COREAD progression too.

### Networking analysis of RNA-Binding proteins vs. colorectal drivers

**FIGURE 4:**
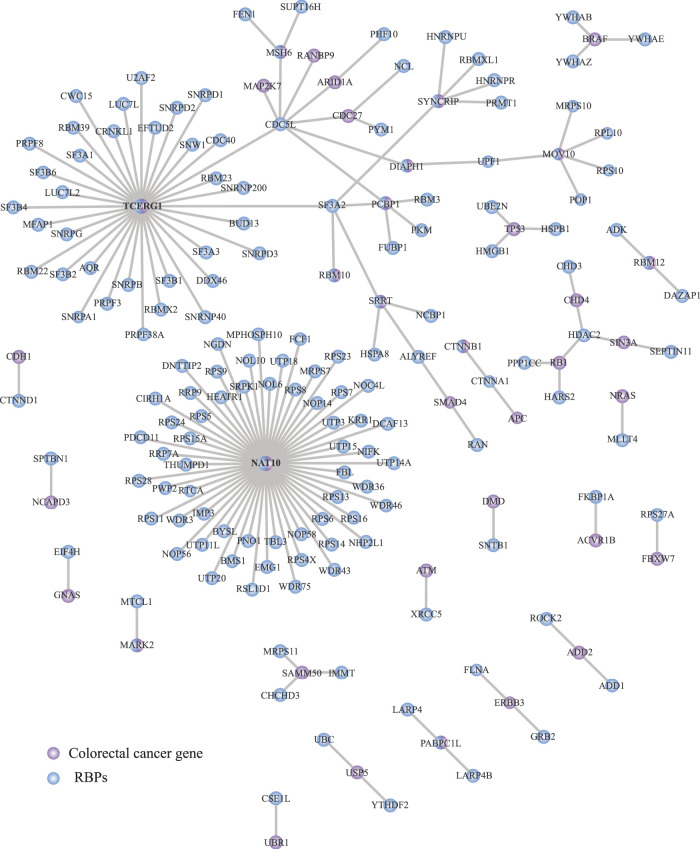
Interaction network between RBPs and colorectal cancer driver proteins. STRING portal (Szklarczyk et al., 2019) was used to analyze protein-protein interactions obtained from experiments and databases. A total of 153 RBPs were identified to be associated with 37 COREAD proteins. Blue = RBPs, purple = COREAD proteins.

Protein-protein interaction (PPIs) networks have been proved to be effective in detecting tumorigenic RNA regulons (Wurth et al., 2016; Indacochea et al., 2021). Thus, we next interrogated the STRING database (Szklarczyk et al., 2019) to understand the relationship between RBPs (*n* = 1,392) and COREAD drivers (*n* = 139) (Repana et al., 2019) and outline key interactions among them. We identified 37 COREAD proteins interacting with 153 RBPs (Figure 4; Supplementary Table S10). The interactions were obtained from experiments and databases using a highest confidence threshold (interaction score = 0.9). As shown in Figure 4, we detected two main interaction networks around TCERG1 and NAT10. Interestingly, these proteins not only have the ability to bind RNA but also, they were catalogued as COREAD drivers. Interestingly, TCERG1 binds to CDCL5 which in turn binds to five COREAD drivers.

### RNA-binding proteins expression levels in colon and rectal tissues

**FIGURE 5:**
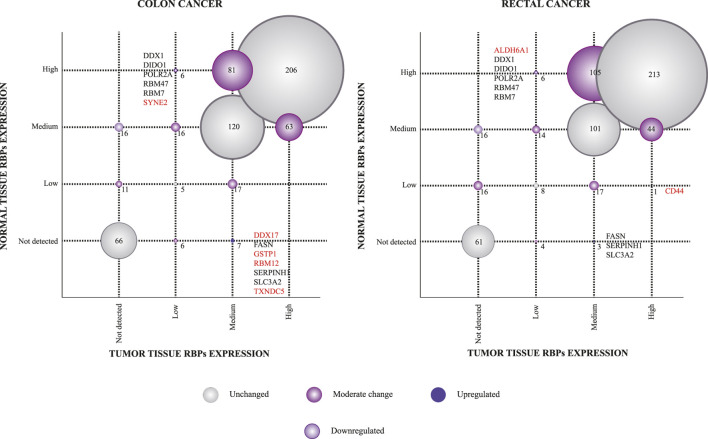
Immunohistochemical protein expression profiles of RNA-binding proteins between healthy and tumoral tissues. Scatterplots comparing RBPs immunohistochemical levels between normal and tumoral tissues are presented. The size of the circle is associated with the number of RBPs found in each correlation. RBPs in red are those which are unique for each type of cancer.

The Human Protein Atlas (HPA) constitutes a large-scale resource to study antibody-based protein expression patterns in human tissues (Uhlén et al., 2015; Thul et al., 2017; Uhlen et al., 2017). We, therefore, used this tool to identify differentially expressed RBPs between tumoral and normal colon and rectal tissues. Thus, we compared protein immunohistochemical levels (not detected, low, medium, and high) of 608 available RBPs in COAD and 609 RBPs in READ tissues. We detected 211 in colon and 226 in rectum RBPs having at least one variation level (e.g., not detected to low or medium to high) between malignant and healthy colon and rectal tissues, respectively (Figure 5; Supplementary Tables S11, S12).

To detect highly altered RBPs, we next categorized these proteins as up or downregulated based on a two-variation level difference. As a result, we found seven upregulated RBPs (DDX17, FASN, GSTP1, RBM12, SERPINH1, SLC3A2, and TXNDC5) and six downregulated (DDX1, DIDO1, POLR2A, RBM47, RBM7, and SYNE2) in colon tissues. As anticipated, our strategy identified well-known COREAD proteins, such as DDX17 (Li X. N. et al., 2018), FASN (Yu et al., 2020) or GSTP1 (Sameer et al., 2012), validating our analysis. It is noteworthy to mention that RBM12 and RBM7 have never been implicated in COAD or READ before. Regarding rectal tissues, we identified four upregulated (CD44, FASN, SERPINH1, and SLC3A2) and six downregulated RBPs (ALDH6A1, DDX1, DIDO1, POLR2A, RBM47, and RBM7). Also, several of these proteins have been previously studied in COREAD as ALDH6A1 (Li X. et al., 2022) or DDX1 (Tanaka et al., 2018). Interestingly, CD44, a well-established COREAD protein (Herrlich et al., 1995; Wielenga et al., 2000), was upregulated only in rectal tumors showing its potential to distinguish rectal from colon carcinomas.

### Identification of RNA binding proteins involved in COAD and READ cell survival

**FIGURE 6:**
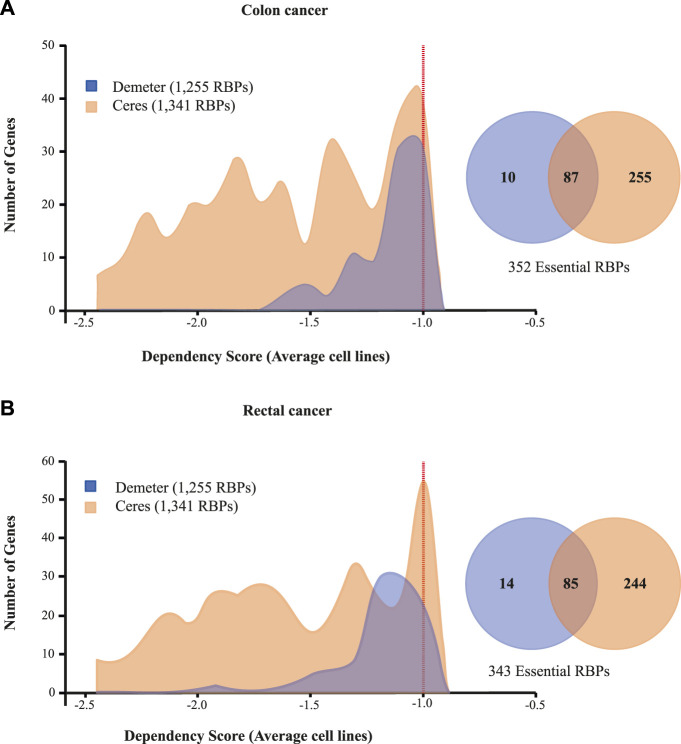
RBPs **(A)** colon and **(B)** rectal cancer dependencies. Dependency scores of 1,255 (DEMETER2; Tsherniak et al., 2017; McFarland et al., 2018) and 1,341 RBPs (CERES; Meyers et al., 2017) from 89 colon and 13 rectal cancer cell lines are presented.

To identify essential RBPs for COAD and READ cell survival, we interrogated two large-scale CRISPR-Cas9 (CERES; Meyers et al., 2017) and RNAi (DEMETER2; Tsherniak et al., 2017; McFarland et al., 2018) loss-of-function screens using the DepMap portal (https://depmap.org/portal/). (Yu et al., 2016) CERES contains data of 1,341 RBPs in 47 colon and 10 rectal cancer cell lines, while DEMETER2 presents data of 1,255 RBPs in 42 colon and three rectal cancer cell lines. These initiatives calculate a dependency score that represents how vital a gene is to cell survival. A score of 0 indicates non-essentiality, whereas a score of ≤ −1 corresponds to the median of all pan-essential genes. Thus, we identified 352 (87 detected by both screens) and 343 (85 detected by both screens) essential RBPs in colon and rectal tumors, respectively (Figures 6A,B; Supplementary Tables S13–S16). Additionally, Table 3 shows the top five essential RBPs for colon and rectal carcinomas.

**TABLE 3:** CRISPR-Cas9 (CERES; Meyers et al., 2017) and RNAi (DEMETER2; Tsherniak et al., 2017; McFarland et al., 2018) cell lines and top five essential RBPs.

	Ceres			Demeter		
	Cell lines	Mean DepScore	Top 5	Cell lines	Mean DepScore	Top 5
Colon	47	−1.6	RAN, RPL15, SNRPB, HSPE1, and RPL4	42	−1.18	SF3B2, RPL7, SNRPD1, RPL14, and EIF3B
Rectum	10	−1.6	RAN, RPL15, HSPE1, RPL23, and RPS6	3	−1.23	SNRPD1, SF3B2, RPL5, COPB1, and SRSF3

### Prioritization of RNA-binding proteins in colon and rectal carcinomas

**FIGURE 7:**
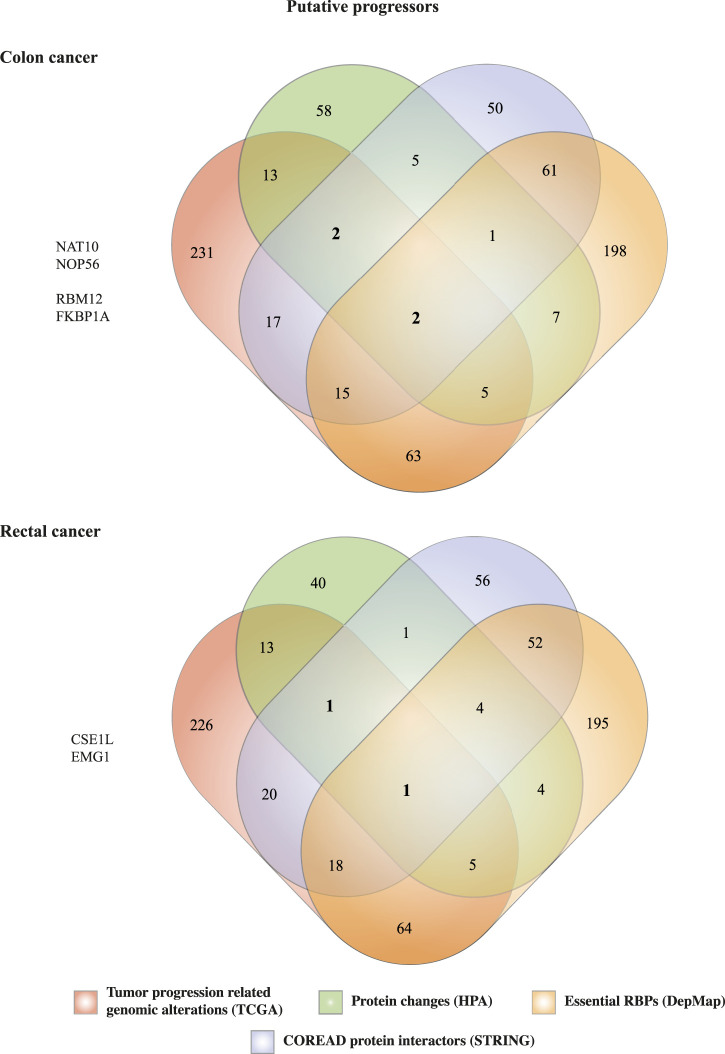
Identification of novel RNA-binding proteins involved in colon and rectal carcinomas progression. Prioritization of RBPs in COAD and READ was based on four cancer-related characteristics 1) high genomic and transcriptomic alterations, 2) interactions with well-known cancer proteins, 3) aberrant protein expression levels compared with normal tissues, and/or 4) essential for tumor survival.

As more and more studies reveal the complex roles of RBPs in cancer progression, new data mining strategies come forward to narrowed down the identification of tumorigenic RBPs (Xing et al., 2021; Chen et al., 2022). To identify potential colon and rectal cancer tumor progressors RBPs, we next used our previously published multidata integration strategy (Figure 1) (García-Cárdenas et al., 2022). Thus, we merged our previous results as follows: 1) first quartile of most genomic and transcriptomic altered RBPs (*n* = 348) concerning tumor progression-related alterations (mRNA upregulation + genomic amplification + gene mutations + fusions), 2) 153 RBPs presenting PPis with colorectal proteins, 3) 93 (colon) and 69 (rectum) RBPs having moderate or upregulated immunohistochemical variation, 4) 352 (colon) and 343 (rectum) essential RBPs (Figure 7).

In COAD, we identified two RBPs (NAT10 and NOP56) having all four tumor-associated characteristics presented in this study. NAT10 has already been implicated in colorectal cancer in several studies (Zhang et al., 2014; Liu et al., 2016; 2019; Cao et al., 2020), while NOP56 has been found differentially expressed in COREAD by a different data mining approach (Liang et al., 2021). Also, we were able to retrieve two other RBPs (RBM12 and FKBP1A) that were not essential for tumor cell survival, but they could be implicated in any other hallmark of cancer. For instance, FKBP1A overexpression has been correlated with apoptosis inhibition in prostate cancer (Leng et al., 2020); we did not find any reports of its involvement in COREAD. On the other hand, RBM12 has been found to be hypermutated in 619 COREAD tumors (Giannakis et al., 2016), but no further experimentation has been performed.

Similarly, we prioritized two RBPs in rectal cancer, CSE1L which presents all the above-mentioned characteristics, and EMG1 that was not essential for tumor cell survival. CSE1L has been related to colorectal cancer before (Sillars-Hardebol et al., 2012; Tai et al., 2013; Pimiento et al., 2016; Xu et al., 2020), while EMG1 has been poorly studied. Interestingly, NOP56, NAT10, and CSEL1 have been classified as common essential by DepMap survival algorithm. Thus, therapeutic targeting of these RBPs could have a greater impact in cell survival due to their implications in RNA-dependent basic cellular processes.

**FIGURE 8:**
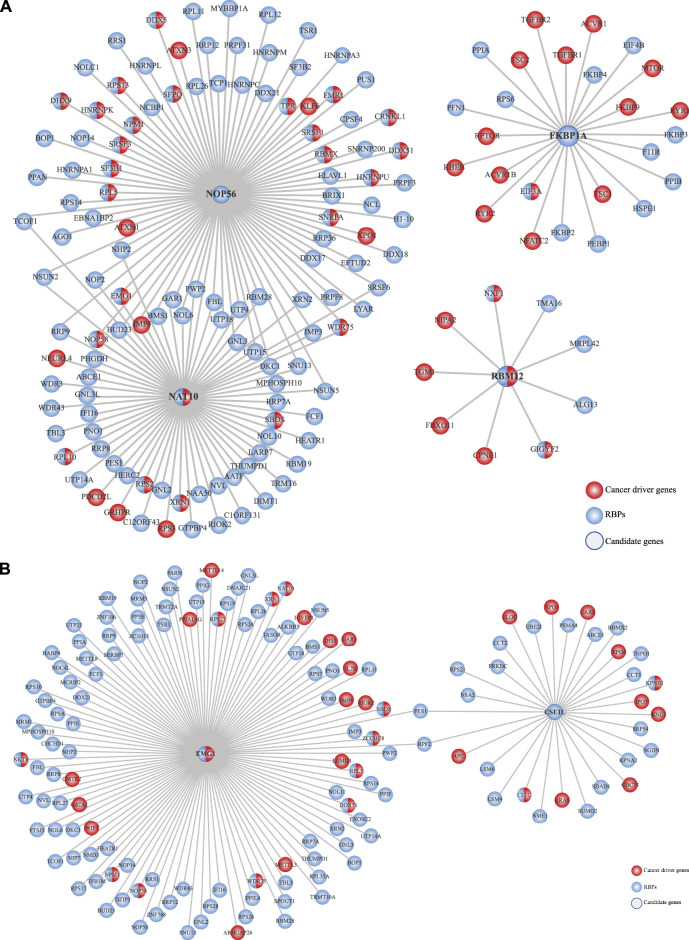
Prioritized RBPs correlate with cancer genes. Previously prioritized RBPs in **(A)** colon cancer (NAT10, NOP56, RBM12, and FKBP1A) and **(B)** rectal cancer (CSE1L and EMG1) were correlated with cancer genes by networking analysis using the HumanNet v3 database (Kim et al., 2022).

Finally, we intended to understand how these prioritized RBPs could correlate with cancer in general terms. To that end, we used the HumanNet v3 (Lee et al., 2011; Hwang et al., 2019) to generate networks where only cancer genes and RBPs were considered. We observed intricated connections between these prioritized RBPs with several cancer genes and other RBPs, showing their potential to promote cancer (Figures 8A,B; Supplementary Tables S17, S18).

### RBPs mRNA expression levels as determinants of clinical outcomes

**FIGURE 9:**
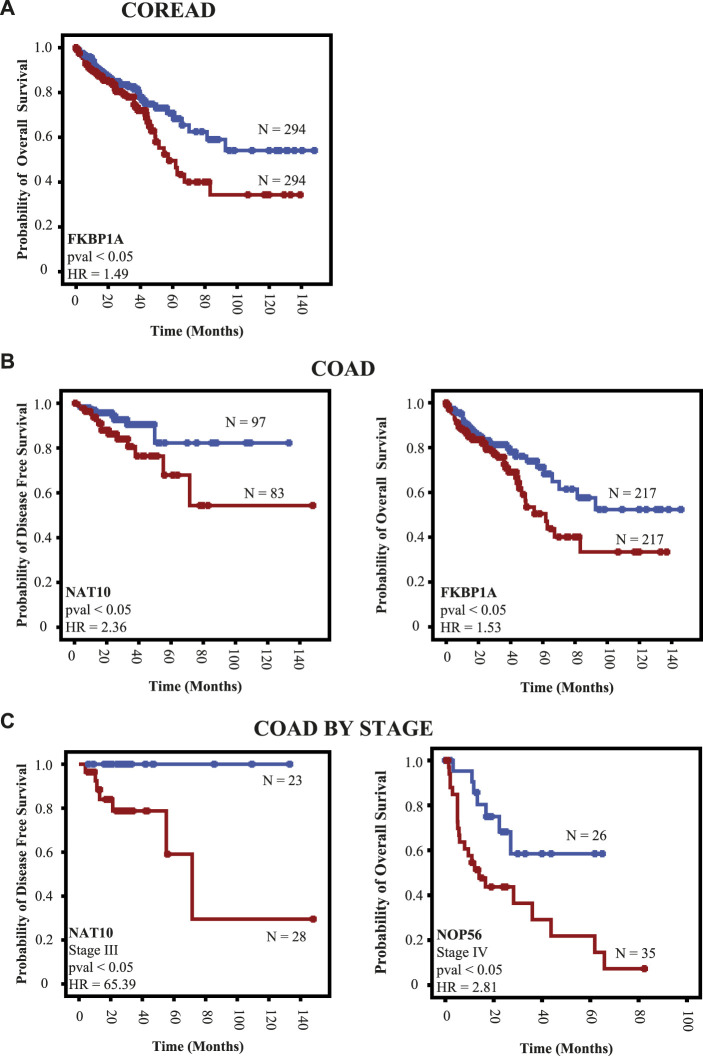
RBPs expression as a determinant of clinical outcome. We interrogated TCGA data using as a cutoff point the median of mRNA expression. **(A)** Prognosis in COREAD population **(B)** prognosis in colon cancer patients regardless the stage, and **(C)** colon cancer patients by stage. Red and blue lines indicate downregulation and upregulation mRNA, respectively.

To explore the clinical relevance of RBPs mRNA expression (upregulation vs. downregulation) in COREAD, COAD, and READ patients, we have interrogated TCGA, PanCancer Atlas (Hoadley et al., 2018) database for mRNA expression of prioritized RBPs (NAT10, NOP56, RBM12, FKBP1A, CSE1L, and EMG1) in those populations and calculated several clinical aspects (OS and DFS). In COREAD population, we only found FKBP1A expression is related with an adverse outcome in overall survival (OS; *p* < 0.05) (Figure 9A).

Due to the fact that colon and rectum cancer are different clinical entities (Paschke et al., 2018), we have explored whether these relevant RBPs could predict clinical outcomes. In COAD patients, NAT10 and FKBP1A are related with adverse outcome in DFS and OS, respectively (*p* < 0.05; Figure 9B). We also found that NAT10 is related with poor outcome in DFS of stage III COAD patients (*p* < 0.05; Figure 9C). Moreover, high expression of NOP56 in primary tumors are related with bad outcome in OS of stage IV COAD patients (*p* < 0.05; Figure 9C). No other clinical outcomes were predicted by RBPs mRNA expression in COREAD, COAD, and READ patients.

## Discussion

The development and implementation of multi-omics approaches along with modern bioinformatic technologies have provided new insights in COREAD biology (Chierici et al., 2020; Yin et al., 2020; Heo et al., 2021). In that respect, several studies have attempted to molecularly characterize COREAD tumors (Schlicker et al., 2012; Budinska et al., 2013; De Sousa E Melo et al., 2013; Marisa et al., 2013; Sadanandam et al., 2013; Roepman et al., 2014). Guinney et al., by integrating several subtyping algorithms, proposed the Consensus Molecular Subtypes of colorectal cancer to establish a baseline for clinical decision making (Guinney et al., 2015). Nevertheless, according to Paschke et al., COREAD has been treated as one entity when several clinical and molecular aspects (e.g., epidemiology, carcinogenic risk, molecular carcinogenesis, etc.) indicate the contrary (Paschke et al., 2018). Consequently, Paschke et al., suggested separating COREAD into COAD and READ, implementing a new perspective to discover novel biomarkers for both cancer types, which holds the potential to improve subtype-based clinical interventions (Paschke et al., 2018). In this regard, RBPs as emerging regulators of cancerous processes (Wurth and Gebauer, 2015; Corrado et al., 2016; Hentze et al., 2018; Kang et al., 2020; Zhang et al., 2020; Chen et al., 2022), could fulfill this need.

In this work, we used our previously published multidata integration strategy to prioritized tumorigenic RBPs in COAD and READ separately (García-Cárdenas et al., 2022). First, we determined genomic and transcriptomic alteration profiles of RBPs in COAD and READ patients. As is shown in Figure 2A, most of the alterations were found in mRNA levels: mRNA upregulation (64% in colon and 62% in rectum) followed by mRNA downregulation (15% in colon and 20% in rectum). Even though genomic and transcriptomic alteration profiles of COAD and READ were similar, we found significant differences among alteration types (Figure 2B) and the level of up and downregulation (Figure 2C), supporting Paschke *et al.*, findings at least regarding RNA metabolism (Paschke et al., 2018). These results also agree with a comprehensive transcriptomic analysis performed by Zhang *et al.*, in which RBPs are predominantly upregulated across cancer types (Zhang et al., 2020). These alterations will probably influence key post-transcriptional processes involved in COAD and READ development.

Despite the genomic and transcriptomic alteration similarities among the subtypes of both carcinomas, most altered RBPs in each subtype differed (Figures 3A,B). At least in READ, most of the highly altered RBPs are unique for this type of cancer, e.g., TSR1, TSR1, DYNC2H1, DDX55, CPSF7, CAND1, LARP4, ZCCHC3, and LSM14B and they have never been associated with COREAD. These findings support Paschke *et al.*, suggestion (Paschke et al., 2018), several RBPs and maybe not only RBPs could have been ignored when we studied as COREAD but, when they are separated in COAD and READ, new putative biomarkers are discovered.

Unlike subtypes, we observed dissimilar patterns of RBPs genomic and transcriptomic alterations among stages in colon and rectal tumors. Stage II was the most altered in COAD, whereas in READ the most altered one was stage I (Figures 3C,D; Table 2). Despite these differences, we found proteins that were constantly altered in all stages. For example, STAU1 and YTHDF1 were highly altered across stages in both carcinomas. Contrary to STAU1, YTHDF1 has previously been associated with COREAD development (Bai et al., 2019; Chen P. et al., 2021; Yan et al., 2021). Interestingly, STAU1 has been related with pancreatic cancer (Marcellus et al., 2021) and its misregulation impacts cell cycle regulation (Bonnet-Magnaval and DesGroseillers, 2021), showing the potential of this protein to also be involved in COREAD progression.

Networking analysis has been shown to be a powerful tool to identify tumorigenic proteins in cancer (Wurth et al., 2016; Indacochea et al., 2021; García-Cárdenas et al., 2022). Similarly, PPIs between RBPs and COREAD drivers allowed us to determine two functional modules, such as NAT10 and TCERG1, which were central elements (Figure 4). These RBPs have also been catalogued as COREAD drivers. In fact, NAT10 suppresses tumor proliferation by activating p53; in COREAD, NAT10 activity is decreased resulting in p53 malfunction and, therefore, uncontrollable cell division (Liu et al., 2016). Concerning TCERG1 subnetwork, we observed intricated connections between RBPs and COREAD drivers. For example, TCERG1 interacts with CDC5L which in turn connects with six COREAD drivers (MAP2K7, RANBP9, ARID1A, CDC27, MSH6, and DIAPH1). Interestingly, CDC5L has been related to other cancer types, such as prostate cancer (Li X. et al., 2018) and osteosarcoma (Lu et al., 2008).

We next examined protein immunohistochemical levels (the Human Protein Atlas database) to identify differentially expressed RBPs in colon and rectal tumor tissues (Pontén et al., 2008). In fact, immunohistochemistry (IHC) is a widely used approach in histopathology for cancer diagnosis. Thus, we found DDX17, GSTP1, RBM12, and TXNDC5 to be overexpressed only in COAD, while CD44 is exclusively upregulated in READ. Similarly, we found SYNE2 and ALDH6A1 to be exclusively downregulated in COAD and READ, respectively. Further IHC studies should be performed to address their potential as diagnostic biomarkers of colon and rectal tumors, separately. As anticipated, our strategy also identified well-known COREAD proteins (Figure 5), such as DDX17 (Li X. N. et al., 2018), ALDH6A1 (Li X. et al., 2022), DDX1 (Tanaka et al., 2018), FASN (Yu et al., 2020), and GSTP1 (Sameer et al., 2012), validating our analysis. It is noticeable to mention that RBM12 and RBM7 have never been implicated in COAD or READ before.

Then, we explored RBPs-based COAD and READ cell dependencies by interrogating two large-scale loss-of-function screens CERES (Meyers et al., 2017) and DEMETER2 (Tsherniak et al., 2017; McFarland et al., 2018). In colon we found 352 essentials RBPs, while in READ we identified 343 RBPs (both CRISPR-Cas9 and RNAi methods included) (Figures 6A,B). In other words, 25% of all known RBPs are essentials for oncogenic cells survival, unsurprisingly given the crucial role of RBPs in RNA metabolism. In Table 3, we listed the top five essentials RBPs in both types of cancer based on the DepScore. The same scenario of the previous analyses is repeated, we obtained RBPs that have been related to COREAD (e.g., SF3B2, RPL7, and SNRPD1), and others that have not (e.g., COPB1) (Supplementary Tables S13–S16) (Boleij et al., 2010; Fijneman et al., 2012; Xu et al., 2021).

Compelling studies have shown the potential of RBPs to promote cancer development. RBPs are widely altered in cancer cells, control hundreds to thousands RNAs, and interact with cancer driver proteins (Wurth and Gebauer, 2015; Hentze et al., 2018; García-Cárdenas et al., 2019). With that in mind, we reasoned that the integration of our previous analyses could narrow down the identification of potential COAD and READ RBPs. Our data mining strategy (Figure 1) allowed us to identify four proteins in COAD (NAT10, NOP56, RBM12, and FKBP1A) and two in READ (CSE1L and EMG1) (Figure 7). NAT10 and CSE1L have already been involved in COREAD (Sillars-Hardebol et al., 2012; Tai et al., 2013; Zhang et al., 2014; Liu et al., 2016; 2019; Pimiento et al., 2016; Cao et al., 2020; Xu et al., 2020). NOP56 and RBM12 were already identified in COREAD by different data mining approaches (Liang et al., 2021), validating our results. Additionally, to the best of our knowledge, no prior studies have associated FKBP1A and EMG1 with cancer before. To highlight their relevance in cancer, we interrogated the HumanNet v3 and found that NOP56, RBM12, FKBP1A and EMG1 are highly interconnected with cancer genes and other RBPs, showing their potential to form tumorigenic RNA-regulons (Figures 8A,B).

Finally, we explored the clinical implications of these prioritized RBPs. Upregulation of FKBP1A, NAT10, and NOP56 mRNA expression could predict clinical outcomes in COREAD and COAD patients. Similarly, mRNA upregulation of NAT10 and NOP56 are related with poor outcomes depending on COAD staging. This is clinically relevant since COAD therapy is defined by stage (Argilés et al., 2020; Cervantes et al., 2022). Stage II COAD patients are not always candidate of adjuvant therapy. However, adjuvant chemotherapy is a relevant treatment for stage III COAD patients because it decreases the risk of relapse (Argilés et al., 2020). In daily clinical practice there are no specific biomarker to predict a relapse. According to our results, NAT10 mRNA upregulation is related with adverse outcome in DFS of stage III COAD patients. Similarly, NOP56 overexpression in primary tumors is associated with poor prognosis in DFS and OS of stage IV COAD patients. These results highlight the clinical relevance of FKBP1A, NAT10, and NOP56. Despite these promising findings, a high number of patients are needed to validate these results in specifical clinical scenarios.

In summary, we analyzed and integrated data from 488 COAD and 155 READ patients, 102 cancer cell lines, more than 15,000 immunostainings, and ∼10,000 raw associations between RBPs and cancer genes to unravel new RBPs involved in COAD (NOP56, NAT10, RBM12, and FKBP1A) and READ (EMG1 and CSE1L). Further analyses allowed us to identify potential clinical applications of FKBP1A, NAT10, and NOP56 as biomarkers of specific outcomes.

## Data Availability

The datasets presented in this study can be found in online repositories. The names of the repository/repositories and accession number(s) can be found in the article/Supplementary Material.
